# Coronary Artery Fistula Presenting as Unstable Angina Pectoris in Patients with Antiphospholipid Syndrome

**DOI:** 10.1155/2013/708947

**Published:** 2013-08-19

**Authors:** Şerafettin Demir, Ceyhun Yucel, Mucahit Tufenk, Aydin Rodi Tosu, Murat Selcuk, Abdi Bozkurt

**Affiliations:** ^1^Adana State Hospital, Department of Cardiology, 01270 Adana, Turkey; ^2^Reyhanli State Hospital, Department of Cardiology, Reyhanli, Hatay, Turkey; ^3^Kızıltepe State Hospital, Department of Cardiology, Kızıltepe, Mardin, Turkey; ^4^Van Education and Research Hospital, Department of Cardiology, Van, Turkey; ^5^Çukurova University, School of Medicine, Department of Cardiology, 01330 Adana, Turkey

## Abstract

The cardiovascular system is one of the primary targets in patients with antiphospholipid syndrome. The valves are the most frequently affected. Atherosclerosis and coronary thrombosis are also seen. The risk of acute coronary syndrome is 10 times higher in patients with APS. We present an APS patient case who was hospitalized with acute coronary syndrome and who was later found to have coronary artery fistula.

## 1. Case Report

**Figure 1:**
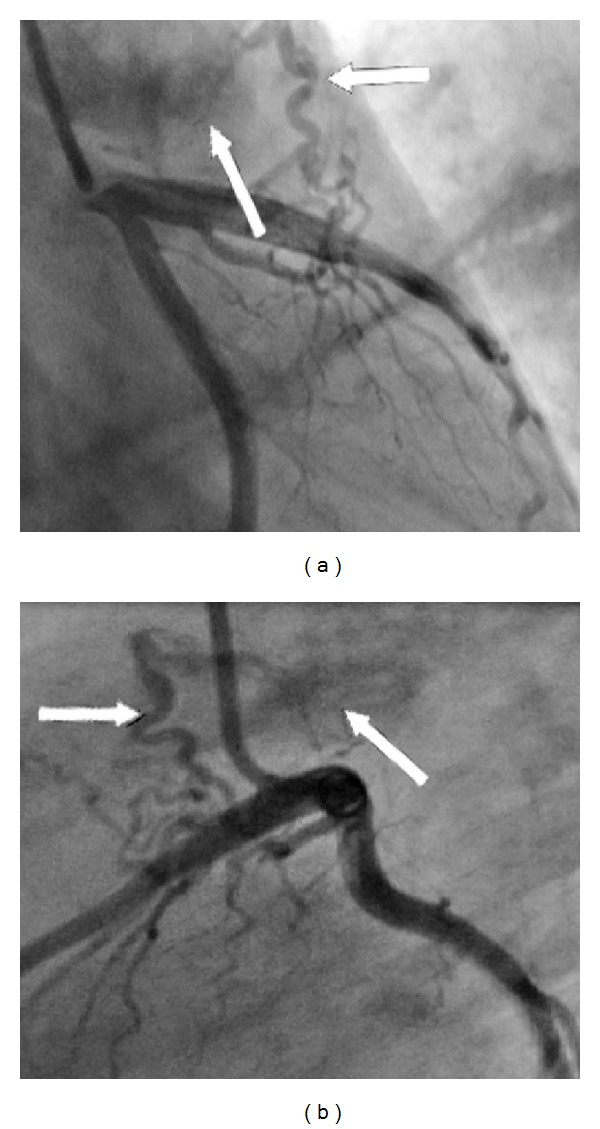
Two branches originating from left anterior descending coronary artery (LAD) and the first diagonal (DI) are combining with a joint path and fistulizing to the pulmonary artery. (a) Right anterior oblique caudal. (b) Lateral view. Thin arrow: fistula; bold arrow: pulmonary artery.

Thirty-six years old female patients applied to emergency service with exercise-related chest pain. The patient was hospitalized with a diagnosis of acute coronary syndrome. Her physical examination, electrocardiogram, and cardiac markers were normal. Her serum antiphospholipid antibody level was increased. She was diagnosed with antiphospholipid syndrome seven years ago. She was treated for hypertension for five years. There were no coronary atherosclerotic lesions at the coronary angiography, but two thin branches from the left anterior descending artery and first diagonal artery were seen to be joined and fistulized to the pulmonary artery (Figures 1(a) and 1(b)). Transcatheter coil embolization was planned for the patient, but the patient did not accept the procedure. She was discharged with medical treatment. 

## 2. Discussion 

The heart is one of the primary target organs affected by antiphospholipid syndrome. Cardiac valves are the most frequently affected, and thickening of valves is usually seen as valvular lesions. There is usually fibrin deposition, vascular proliferation, fibrosis, and calcification in the histopathologic examination of valves [1–5]. Thrombosis and atherosclerosis of coronary arteries is another type of cardiac involvement. Patients with antiphospholipid syndrome have more than 10 times increased risk of acute myocardial infarction [5]. The correlation between antiphospholipid antibodies and thrombosis, acute myocardial infarction, and cerebral vascular events was represented by the prospective studies [4, 5]. Although there are a number of mechanisms for the development of acute coronary syndrome, atherosclerosis and hypercoagulability are prominent factors [5]. In our case, due to the presence of APS, acute coronary syndrome was first associated with existing disease; however, after coronary angiography, we detected coronary artery fistula with no coronary artery stenosis [3]. Coronary arteriovenous fistula is seen in one per fifty-thousand live births and is usually detected in childhood and young adulthood [6]. The incidence of coronary artery fistulas have been reported as 0.2–0.8% in angiography series [6, 7]. Patients with coronary artery fistula are usually asymptomatic. Depending on the severity of shunting, dyspnea, fatigue, orthopnea, angina pectoris, endocarditis, arrhythmias, stroke, myocardial ischemia, or infarction can be monitored [8, 9]. In our case, the main symptom was chest pain compatible with angina pectoris. 

The most common finding in physical examination is a continuous murmur heard in the left parasternal area extending to the apex. Nonspecific electrocardiographic changes, cardiomegaly, and increased pulmonary vascular appearance in chest X-rays have been observed in half of the cases [7–10]. Physical examination of our case was normal. Two-dimensional and Doppler echocardiography, cardiac catheterization, and coronary angiography are effective diagnostic methods [10]. Our case was diagnosed by coronary angiography. 

Coronary artery fistulas are usually present at birth but may occur rarely after thoracic trauma. They may originate from each of the three coronary arteries, but right coronary arteries are often the most affected coronary arteries, and fistulas are usually connected with right heart chambers or the pulmonary artery [9, 10]. In the study of Onbasılı et al., 26% of fistulas were connected with the right ventricle, 26% with the right atrium, and 13.8% with the pulmonary artery [10]. Fistulas are most frequently between coronary arteries or their branches and venous system (90%) [8–10]. In most cases, fistulas are small and have minor shunts. These patients are asymptomatic; however, fistulas with a large shunt can be related with congestive heart failure [8]. Myocardial ischemia due to reduced blood flow to the distal part of coronary artery may develop even if there is no coronary atherosclerosis. In our case, the fistula originated from LAD and was connected with the pulmonary artery. It is known that 73% of isolated coronary artery fistulas are symptomatic [9]. In one study, 12% of symptoms were related with congestive heart failure, 4% with myocardial infarction, 1% with rupture, and 3% with bacterial endocarditis. Our case was admitted to hospital with signs of acute coronary syndrome (unstable angina pectoris). Current approaches for treatment include medical treatment, percutaneous embolization, or surgical treatment [9, 10]. Intervention is not indicated in asymptomatic patients with small fistulas, and they can be followed with medical treatment [8]. There is a general consensus on closure of the fistula in symptomatic patients [10]. 

As a result, this case shows us that coronary artery fistulas may lead to acute coronary syndrome, and it should be considered as an etiologic factor in young patients.
